# 2022 FDA TIDES (Peptides and Oligonucleotides) Harvest

**DOI:** 10.3390/ph16030336

**Published:** 2023-02-22

**Authors:** Othman Al Musaimi, Danah Al Shaer, Fernando Albericio, Beatriz G. de la Torre

**Affiliations:** 1 Department of Chemical Engineering, Imperial College London, London SW7 2AZ, UK; 2 Department of Medicinal Chemistry, Evotec (UK) Ltd., Abingdon OX14 4R, UK; 3 School of Chemistry and Physics, University of KwaZulu-Natal, Durban 4001, South Africa; 4 CIBER-BBN, Networking Centre on Bioengineering, Biomaterials and Nanomedicine, Department of Organic Chemistry, University of Barcelona, 08028 Barcelona, Spain; 5 KRISP, College of Health Sciences, University of KwaZulu-Natal, Durban 4001, South Africa

**Keywords:** drugs, FDA, oligonucleotides, peptides, vutrisiran, gadopiclenol, tirzepatide, lutetium (^177^Lu) vipivotide tetraxetan, terlipressin

## Abstract

A total of 37 new drug entities were approved in 2022; although that year registered the lowest number of drug approvals since 2016, the TIDES class consolidated its presence with a total of five authorizations (four peptides and one oligonucleotide). Interestingly, 23 out of 37 drugs were first-in-class and thus received fast-track designation by the FDA in categories such as breakthrough therapy, priority review voucher, orphan drug, accelerated approval, and so on. Here, we analyze the TIDES approved in 2022 on the basis of their chemical structure, medical target, mode of action, administration route, and common adverse effects.

## 1. Introduction

**Figure 1:**
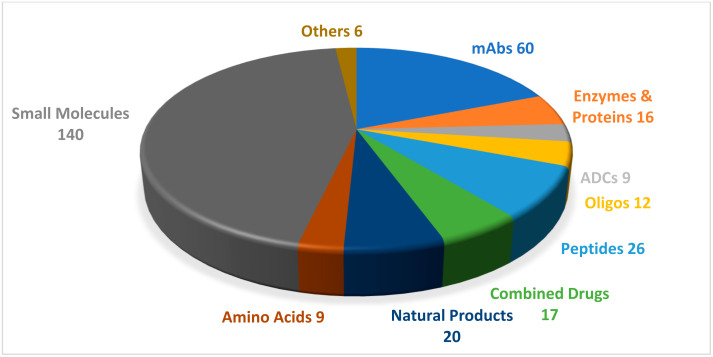
A total of 315 new drugs were approved by the Food and Drug Administration (FDA) between 2016 and 2022 [1]. Adapted with permission from Ref. [1]. Copyright 2022, copyright MDPI. mAbs, monoclonal antibodies; ADCs, antibody-drug conjugates; Oligos, oligonucleotides.

A total of five TIDES (four peptides and one oligonucleotide) were authorized by the United States Food and Drug Administration (FDA) in 2022 [1]. This harvest accounts for 14% of the total number of approvals of new drug entities that year (which amounted to 37; Figure 1). For the purpose of this series of reviews, we define peptide-based drugs as compounds that carry at least two amino acids in their structure, that are synthesized mainly by chemical methods, and whose purity has been assessed by analytical methods.

TIDE authorizations in 2022 were the lowest in number on record in the last five years [2]. This figure could be attributed to the COVID-19 pandemic, as the pharmaceutical industry had to channel resources into the development of vaccines and drugs against this disease (Table 1).

Of note, three of the peptides that received the green light in 2022 are considered first-in-class—namely, Mounjaro^TM^ (which activates glucagon-like peptide-1 (GLP-1) and glucose-dependent insulinotropic polypeptide (GIP), Terlivaz^TM^ (first-line treatment for hepatorenal syndrome (HRS)), and Pluvicto^TM^ (first targeted radioligand therapy (RLT)) [3]. Therefore, they were assigned fast-track designation by the FDA to accelerate the approval process.

Diverse structures, including linear, disulfide bridge, and peptide mimics, were included in these approvals.

## 2. Oligonucleotides

Only one oligonucleotide-based drug made it to the market in 2022. However, this class of drug has demonstrated enormous potential for the treatment of rare fatal hereditary disorders over the past few years. In addition, these drugs have provided a solid foundation for the development of many other candidates in the pipeline that target a variety of disorders [1]; this is reflected by the magnitude of collaborations and agreements between leading pharmaceutical institutions in this field [4].

### Vutrisiran (Amvuttra^TM^)

**Figure 2:**
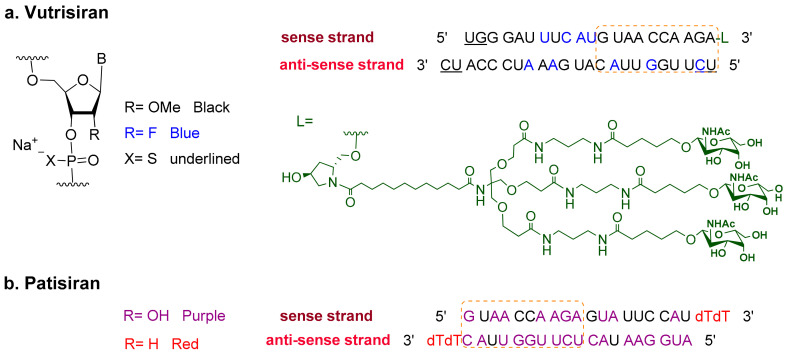
Chemical structure of (**a**). Vutrisiran (Amvuttra^TM^) [6] vs. (**b**) Patisiran (Onpattro^TM^) [11].

Vutrisiran is a short interference RNA (siRNA) that consists of the sodium salt of two chemically modified strands (sense and antisense). The sense strand comprises 21 modified monomers—three of which have 2′-fluororiboso, and the rest of which have a methoxy substituent at the same position. The strand starts with two phosphorothioate linkages at its 5′ terminus and ends at its 3′-terminus with the covalently bonded ligand, L. The driving ligand is a dendrimer with *N*-acetyl galactoseamine pendants that targets the asialoglycoprotein receptor on hepatocytes [5]. In contrast, the 23-mer antisense strand has five 2′-fluoro substituted riboso subunits while the rest are methoxy substituted, in addition to two thioate moieties on the two termini (Figure 2) [4,6].

Vutrisiran is the fourth drug to use the Enhanced Stabilization Chemistry (ESC)-N-GalNAc delivery platform [7], which was first introduced by Alnylam in 2019 [8,9,10]. This platform boosts the pharmacodynamic and pharmacokinetics profiles of liver-targeting drugs [4].

Vutrisiran is used for the treatment of polyneuropathy hereditary transthyretin-mediated amyloidosis in adults (hATTR) [6]. This condition occurs as the result of the accumulation of amyloid fibrils in peripheral nerves and the heart [4,7]. Amyloidosis is caused by the deposition of the misfolded transthyretin protein (TTR), the molecule that is produced by hepatocytes and is responsible for transferring vitamin A and thyroxine [4]. The misfolding of this protein and the consequent deposits are caused by a hereditary mutation in the TTR gene—leading to hATTR—or are present in the wild-type and brought about by aging (wtATTR) [4].

Vutrisiran targets and silences TTR-mRNA, thereby downregulating the faulty protein and its consequent accumulation [7]. It is the second siRNA drug to be approved by the FDA for the treatment of hATTR after patisiran, which was approved in 2018 [12,13]. Both drugs share a 10-mer segment with the same sequence of nitrogenous bases (dashed orange box, Figure 2). However, all the riboso units in vutrisiran are chemically modified. This feature contrasts with patisiran, which contains seven natural RNA nucleosides within the same shared segment. Additionally, the double strands of patisiran are enclosed in liposomal nanoparticles to facilitate its delivery to the liver [11,14].

Vutrisiran is administered subcutaneously from a prefilled syringe at a dose of 25 mg every three months [6,7]. Clinical studies have shown moderate to mild adverse effects in most patients (97.5%) who are treated with vutrisiran, with severe events reported in 15.6% compared to 38.1% of those administered patisiran. These results conclude a good tolerance of vutrisiran [4]. Nevertheless, it reduces the production of TTR protein—responsible for transferring vitamin A—thereby causing a decrease in vitamin A levels. Hence, a daily dose of this vitamin is recommended when night blindness is observed as a side effect. Other adverse effects may appear, including arthralgia and dyspnea [6]. Clinical studies focusing on mATTR patients with cardiopathy [15] or polyneuropathy [16] are ongoing and are estimated to finish in 2025 [15]. Vutrisiran was developed by Alnylam (Cambridge, MA, USA) under the Alnylam–Sanofi agreement for the development of hATTR drugs; it was approved by the FDA on 13 June 2022 [4].

## 3. Peptides

### 3.1. Gadopiclenol (Elucirem^TM^)

**Figure 3:**
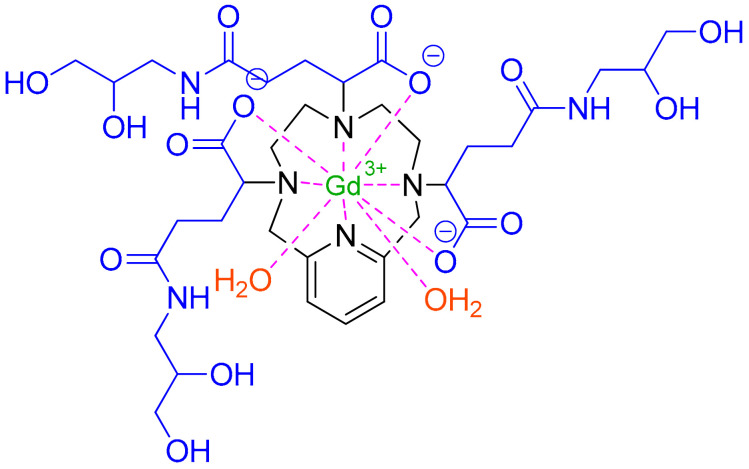
Chemical structure of Gadopiclenol (Elucirem^TM^). Brown: two water molecules that show coordination in solution [17].

Gadopiclenol is a non-ionic Gd^3+^ complex with a pyridine-containing triaza (PCTA) macrocycle (Figure 3, in black) with three ionizable (2,3-dihydroxypropyl)amino)-5-oxopentanoic acid pendants (Figure 3, blue) [17]. The macrocyclic chelator stabilizes the overall complex compared to linear ones [18], with a non-detectable release of Gd^3+^ ions inside the body [17].

Gadopiclenol is a gadolinium-based contrast agent (GBCA) used for magnetic resonance imaging (MRI) to detect lesions in different parts of the body [19,20]. It is an extracellular fluid (ECF) contrast agent and as such, it rapidly leaves the bloodstream and accumulates in extracellular spaces. Tumors are usually associated with abnormal vascularity and thus, they retain higher concentrations of the contrast agent in their vicinity than healthy tissues—thereby facilitating visualization [20].

Gadopiclenol has paramagnetic properties and, therefore, develops a magnetic moment when placed in a magnetic field [19]. Thus, it selectively increases the contrast of MRI imaging in tissues where it accumulates as it affects the protons of nearby water molecules, altering their relaxation times. These variations in signal intensity, which depend on proton densities in different tissues, allow tissue visualization during MRI [19]. Compared to other GBCAs, gadopiclenol shows high relaxivity—thereby allowing the dose of gadolinium to be reduced by half [21].

Gadopiclenol is administered intravenously in single-dose prefilled syringes and has a half-life of 1.5 h. Clinical studies have shown mild adverse events post-injection in 50% of recipients; these did not differ to the adverse events reported in placebo recipients [22], while in another study, adverse effects were reported in 14.6% of gadopiclenol recipients compared to 17.6% of those administered gadobutrol [23]. Most of these effects were mild, thereby indicating the acceptable safety of the drug. The main adverse effects reported included injection site pain, headache, nausea, injection site warmth and coldness, dizziness, and localized swelling [19]. However, as gadopiclenol is excreted unchanged in the urine, it may cause an increased risk of nephrogenic systemic fibrosis (NSF) in patients with impaired elimination of these agents [19,20].

Gadopiclenol was developed by Guerbet (Villepinte, France) [24]. It was approved by the FDA on 21 September 2022 [24].

### 3.2. Tirzepatide (Mounjaro^TM^)

**Figure 4:**
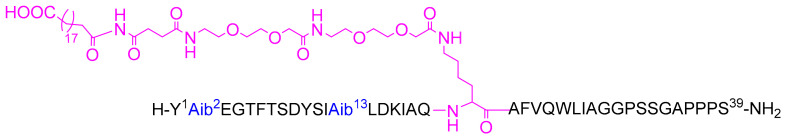
Chemical structure of tirzepatide (Mounjaro^TM^). Blue: Aminoisobutyric acid Aib unnatural amino acid; Pink: C20 fatty acid di-acid moiety.

Tirzepatide is a 39-mer peptide amide that comprises a C20 fatty acid di-acid moiety that binds to albumin [25], thus extending its period of action and offering the possibility of weekly administration [26]. It has two unnatural amino acids (Aib) at positions 2 and 13 that enhance its resistance to peptidase [27]. The 1,20-eicosanedioic acid moiety is derived from the ε-amino function of Lys20. Tirzepatide has a molecular weight of 4813.53 g/mol (Figure 4).

Tirzepatide has agonist activity at both receptors—namely, the GIP and GLP-1 receptors [26,28]. It is prescribed as an adjunct to diet and exercise to control blood pressure in type II diabetes. Clinical studies demonstrated the superiority of tirzepatide over the selective GLP-1RA dulaglutide [28]. Tirzepatide has been reported to improve beta-cell function and insulin sensitivity in type 2 diabetes, allowing greater glucose control and weight loss [28]. It has also been demonstrated that tirzepatide outperforms semaglutide (FDA approved in 2017 [29]), as reflected by the mean change in the glycated hemoglobin level from baseline to 40 weeks at a dose of 1 mg [30].

Tirzepatide is administered subcutaneously and has shown adverse effects, including nausea (17–22%), diarrhea (13–16%), reduced appetite, vomiting (6–10%), constipation, abdominal pain, and dyspnea [26,31]. It is worth highlighting that the same adverse effects, to the same extent, have been observed in patients who received semaglutide [30].

Tirzepatide was developed by Eli Lilly and company (Indianapolis, IN, USA) and approved by the FDA on 13 May 2022 [31].

### 3.3. Lutetium ^177^Lu Vipivotide Tetraxetan (Pluvicto^TM^)

**Figure 5:**
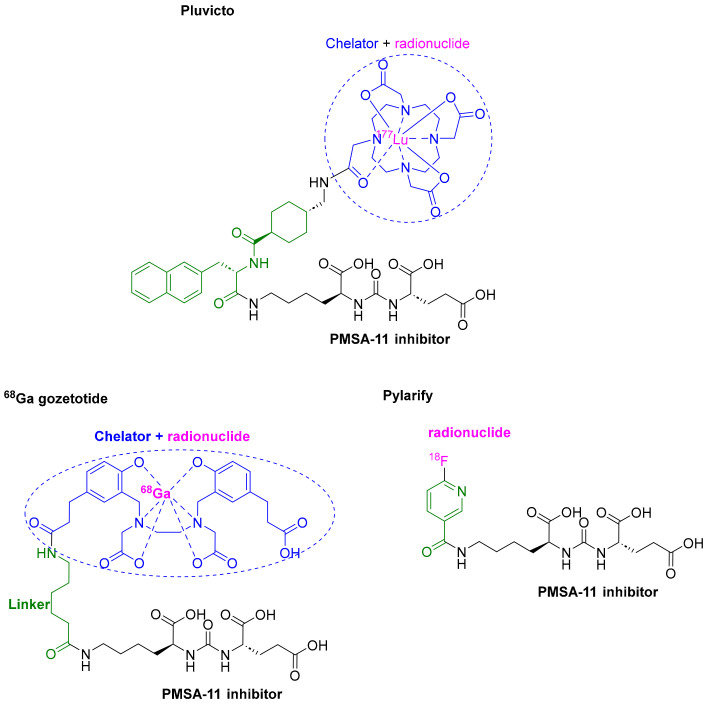
Chemical structure of PSMA-containing drugs ^177^Lu vipivotide tetraxetan (Pluvicto^TM^), ^68^Ga gozetotide, and piflufolastat F18 (Pylarify^TM^).

Previously known as [^177^Lu]Lu-PSMA-617, lutetium ^177^Lu vipivotide tetraxetan is a radioligand therapeutic agent with a molecular weight of 1216.06 g/mol [32]. It comprises a prostate-specific membrane antigen (PSMA)-binding ligand that is a urea-based HO-Glu-NH-CO-NH-Lys-OH (black) bound to a ^177^Lu-labeled DOTA (1,4,7,10-tetraazacyclododecane-1,4,7,10-tetraacetic acid) chelator (blue) through the 2-naphthyl-L-Ala-trans-cyclohexyl linker (green) [1]. After authorization of ^68^Ga gozetotide in 2020 [9] and Pylarify in 2021 [10], ^177^Lu vipivotide tetraxetan is the third drug to be approved that shares the same PSMA-11 binding ligand. The three drugs differ in the radionuclide, chelator (if present), and linker to which the PSMA-11 is attached. The driving ligand in ^68^Ga gozetotide is attached to a lipophilic acyclic HBED-CC (N,N′-bis (2-hydroxy-5-(carboxyethyl)benzyl) ethylenediamine*N*,*N*’- diacetic acid; blue), chelating the ^68^Ga radionuclide (pink) thorough a Lys linker (green) [9]. In contrast, in Pylarify, it is covalently attached to ^18^F radionuclide (pink) through a nicotinyl linker (green) [10] (Figure 5). ^177^Lu vipivotide tetraxetan is the second ^177^Lu-containing drug to receive the green light after the approval of Lutathera^TM^ in 2018 [13].

Lutetium ^177^Lu vipivotide tetraxetan is used to treat prostate cancer after the administration of other therapies (such as androgen receptor (AR) pathway inhibition and taxane-based chemotherapy). It binds to PSMA, a transmembrane protein that is overexpressed in prostate cancer, including castration-resistant prostate cancer (mCRPC). Upon binding, the β-emission of ^177^Lu delivers radiation to PSMA-expressing cells, as well as neighboring ones, and induces DNA damage and subsequent cell death. Hence, ^177^Lu is the active radionuclide in this therapeutic agent. Most importantly, treatment with lutetium ^177^Lu vipivotide tetraxetan does not result in radioligand therapy-induced deterioration of renal function [33].

Lutetium ^177^Lu vipivotide is administered intravenously and has shown adverse effects such as fatigue, nausea, dry mouth, anemia, reduced appetite, and constipation [34].

Serious adverse effects were observed in 36% of patients, whereas 2.8% of patients who had concurrent treatment-related thrombocytopenia experienced fatal adverse reactions [35]. In another study, lutetium ^177^Lu vipivotide tetraxetan was compared with cabazitaxel, which is a type of chemotherapy for the treatment of prostate cancer [36,37]. Thirty-three percent of patients showed adverse effects vs. 53%, respectively [36]. With an overall higher PSA response to lutetium ^177^Lu vipivotide tetraxetan than cabazitaxel, fewer adverse reactions, and no reported deaths attributed to ^177^Lu vipivotide tetraxetan, it is considered a potential replacement for cabazitaxel [36].

Lutetium ^177^Lu vipivotide was developed at DKFZ (German Cancer Research Center) and the University Hospital Heidelberg [32] and was then exclusively licensed to advanced Biochemical Compounds, which in turn licensed it to Endocyte, a Novartis subsidiary (Indianapolis, IN, USA) [37]. Finally, lutetium ^177^Lu vipivotide was approved by the FDA on 23 March 2022 as the first RLT [38].

### 3.4. Terlipressin (Terlivaz^TM^)

**Figure 6:**
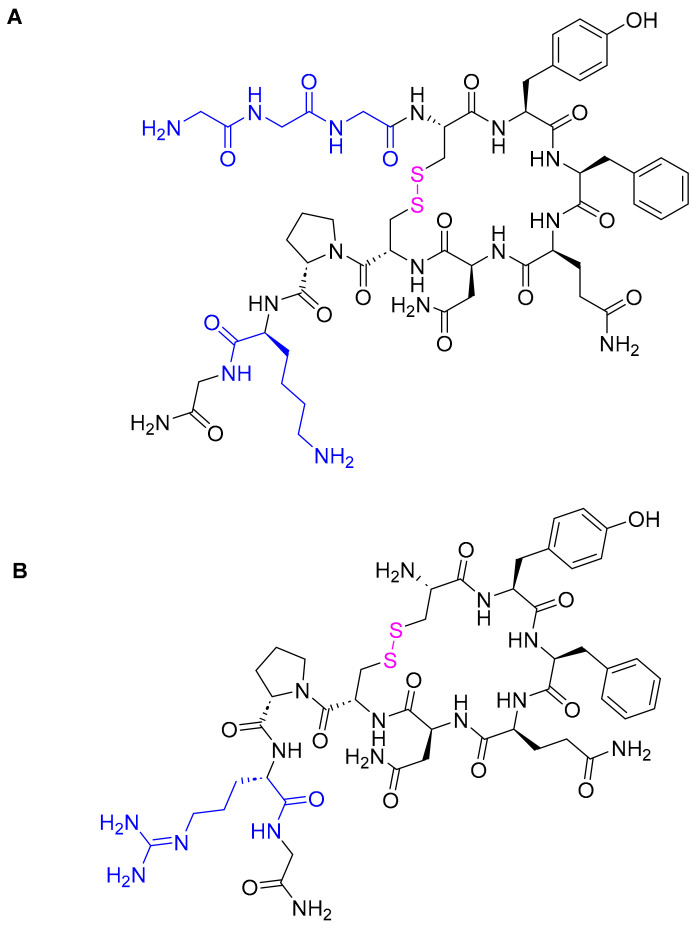
Chemical structure of: (**A**) terlipressin (Terlivaz^TM^). (**B**) Vasopressin. Pink: disulfide bridge. Differences between the two peptides are shown in blue.

Terlipressin comprises nine amino acids—five of which form a macrocyclic peptide via a disulfide bridge, where the remaining four amino acids are pending from the *C*-terminal of Cys. Terlipressin is an analog of vasopressin with several modifications (Figure 6). It is a vasopressin receptor agonist (triglycyl lysine vasopressin) with a molecular weight of 1227.38 g/mol.

Terlipressin increases renal blood flow in hepatorenal patients by reducing portal hypertension and blood circulation and increasing the effective and mean arterial volume and pressure, respectively [39]. It is also increasingly considered a promising medication for the life-threatening septic shock condition [40], as well as a safe and effective treatment for acute oesophageal variceal bleeding [40]. Clinical trials demonstrated that terlipressin resulted in a decrease in the levels of serum creatinine (SCr) to <1.5 mg/dL for at least 48 h by day 14 without dialysis, relapse of HRS type 1, or any reported deaths [41]. Furthermore, it has reduced mortality, failure of hemostasis, and incidence of emergency procedures for patients with uncontrolled bleeding or rebleeding cases [41]. Terlipressin has more favorable pharmacokinetics and selectivity than vasopressin. Furthermore, its longer half-life allows for fewer intravenous injections [42].

It is the first FDA-approved drug prescribed to improve kidney function in adults with HRS [39,43]. It is administered intravenously and has shown adverse effects, including abdominal pain, nausea, respiratory failure diarrhea, and dyspnea [39].

There was no significant difference between terlipressin and any other controlled groups in terms of adverse reactions [41]. Six deaths out of 798 patients who received terlipressin vs. five out of 811 patients in the control group were reported [41]. However, these deaths were not directly related to the treatment [41]. It is worth highlighting that several patients relapsed after terlipressin therapy withdrawal [44].

Terlipressin was developed by Mallinckrodt Pharmaceuticals (Blanchardstown, Dublin, Ireland) and approved by the FDA on 14 September 2022 [45].

## 4. Conclusions

The FDA approved a total of 53 drugs in 2020 [9] vs. only 37 in 2022 [1]—the latter registering the smallest number of authorizations since 2016. Nonetheless, a total of five drugs under the TIDES class received the green light in 2022. Three out of the four peptides approved were first-in-class. The new approvals showed excellent performance, which has definitely brought them to the market as new and/or alternative treatments to existing therapeutics.

These continuous approvals of the TIDES class are attributed to advancements in synthetic methodologies. The tight link and synchronization between the medicinal chemistry discipline and the pharmaceutical industry have helped in the deployment of new drug families with unprecedented overall pharmacokinetics (PK) and pharmacodynamics (PD). It is worth drawing attention to the scarcity of orally available peptide-based drugs. In this regard, new strategies are required to ensure their efficient deployment.

With time, we will undoubtedly witness new peptide and oligonucleotide families with superior stability and efficacy, and also perhaps more orally available analogs, which will enhance the comfort of treatments for patients.

## Figures and Tables

**Table 1:** Summary of FDA-approved TIDES in 2022.

No.	Active Ingredient (Trade Name)	Indication	Therapeutic Target	Administration Route
Oligonucleotides				
1	Vutrisiran (Amvuttra^TM^)	To treat polyneuropathy of hereditary transthyretin-mediated amyloidosis.	TTR mRNA	Subcutaneous
Peptides				
2	Gadopiclenol (Elucirem^TM)^	To detect and visualize lesions, together with MRI, with abnormal vascularity in the central nervous system and the body.	Extracellular fluids in tissues with abnormal vascularity	Intravenous
3	Tirzepatide (Mounjaro^TM^)	To improve blood sugar control in diabetes, in addition to diet and exercise.	GIP and GLP-1 receptors	Subcutaneous
4	Lutetium (^177^Lu) vipivotide tetraxetan (Pluvicto^TM^)	To treat prostate-specific membrane antigen-positive metastatic castration-resistant prostate cancer after other therapies.	PSMA	Intravenous
5	Terlipressin (Terlivaz^TM^)	To improve kidney function in adults with hepatorenal syndrome with rapid reduction in kidney function.	V1 and V2 receptors	Intravenous

GLP-1: glucagon-like peptide-1; GIP: glucose-dependent insulinotropic polypeptide; PSMA: prostate-specific membrane antigen.

## Data Availability

Not applicable.
